# Use of therapeutic caffeine in acute care postoperative and critical care settings: a scoping review

**DOI:** 10.1186/s12871-021-01320-x

**Published:** 2021-03-31

**Authors:** M. Bright, V. Raman, K. B. Laupland

**Affiliations:** 1 Department of Anaesthetics, Princess Alexandra Hospital, Queensland and Faculty of Medicine, The University of Queensland (UQ), Brisbane, Queensland Australia; 2 Department of Intensive Care Services, Royal Brisbane and Women’s Hospital and Faculty of Health, Queensland University of Technology (QUT), Brisbane, Queensland Australia

**Keywords:** Caffeine, Coffee, Intensive care unit, Critical care, Anesthesia, Postoperative period

## Abstract

**Background:**

Caffeine is the most utilised psychoactive drug worldwide. However, caffeine withdrawal and the therapeutic use of caffeine in intensive care and in the perioperative period have not been well summarised. Our objective was to conduct a scoping review of caffeine withdrawal and use in the intensive care unit (ICU) and postoperative patients.

**Methods:**

PubMed, Embase, CINAHL Complete, Scopus and Web of Science were systematically searched for studies investigating the effects of caffeine withdrawal or administration in ICU patients and in the perioperative period. Areas of recent systematic review such as pain or post-dural puncture headache were not included in this review. Studies were limited to adults.

**Results:**

Of 2268 articles screened, 26 were included and grouped into two themes of caffeine use in in the perioperative period and in the ICU. Caffeine withdrawal in the postoperative period increases the incidence of headache, which can be effectively treated prophylactically with perioperative caffeine. There were no studies investigating caffeine withdrawal or effect on sleep wake cycles, daytime somnolence, or delirium in the intensive care setting. Administration of caffeine results in faster emergence from sedation and anaesthesia, particularly in individuals who are at high risk of post-extubation complications. There has only been one study investigating caffeine administration to facilitate post-anaesthetic emergence in ICU. Caffeine administration appears to be safe in moderate doses in the perioperative period and in the intensive care setting.

**Conclusions:**

Although caffeine is widely used, there is a paucity of studies investigating withdrawal or therapeutic effects in patients admitted to ICU and further novel studies are a priority.

**Supplementary Information:**

The online version contains supplementary material available at 10.1186/s12871-021-01320-x.

## Background

Caffeine is the most widely used psychoactive drugs worldwide and has been used therapeutically in anaesthesia, critical care, and pain medicine [1]. Perioperatively, caffeine (≥ 100 mg) provides adjunctive pain relief effects when added to common analgesics [2], and is commonly used in the treatment of post-dural puncture headaches [3]. Postoperatively the use of caffeine after elective colorectal surgery has been recommended to reduce the incidence of postoperative ileus [2, 4]. Caffeine has been used in neonatal intensive care units (ICU) to treat apnoea related syndromes with no long-term adverse effects [5, 6]. However, its effects are less well defined in critically ill adults and therapeutic use must be considered in the context of whether patients are chronic users or not.

Caffeine is a derivates of methylxanthine that acts by inhibiting adenosine receptors and the downstream neurotransmitters (releases norepinephrine, dopamine, and serotonin in the brain, promoting lipolysis and can increase blood catecholamines [7–9]. In infants, caffeine and methylxanthine act on central and peripheral receptors that stimulate the medullary respiratory centre [10]. This has a range of physiologic, cognitive, and psychomotor effects and influences wakefulness and sleep [11–13]. Caffeine has a complex relationship with endothelial cell function, in which it can cause vasodilation by increased intracellular calcium increasing nitric oxide, or vasoconstriction mediated by adenosine antagonism. The effects on the cardiovascular system are seen by mild changes to heart rate and blood pressure, with no consensus in the literature that increased caffeine consumption will increase risk of arrhythmias [14–17]. Abrupt cessation of caffeine in chronic users, such as with fasted patients postoperatively, will affect 10–55% of individuals and may have adverse effects such as increased cerebral blood flow velocity, quantitative electroencephalogram changes and symptoms including headache, drowsiness, decreased alertness, flu like symptoms, nausea/vomiting, and myalgias [18, 19]. Its potential uses to treat withdrawal symptoms, to moderate disturbed sleep-wake cycles, and reduce ICU/postoperative delirium have not been systematically reviewed. Our objective was to conduct a scoping review surrounding the use of caffeine in acute care postoperative and critical care settings in order to summarize the available published evidence and to identify future research priorities.

## Methods

### Data sources

PubMed, Embase, CINAHL Complete, Scopus and Web of Science were searched using the Medical Subject Headings (MeSH) and key words on 1st May 2020 (see Additional file 1: Appendix).

### Study questions

1. What is the evidence for caffeine in the ICU and what is the evidence for caffeine perioperatively?
  - Caffeine withdrawal and administration on the development of postoperative headache or delirium
  - Caffeine withdrawal and administration on induction and emergence from sedation
  - Safety and changes associated with caffeine administration

### Inclusion criteria

Studies of any methodology were included if they investigated the effect or safety of caffeine or caffeine-containing products on hospitalised patients, with a focus on perioperative patients and those admitted to the ICU.

### Exclusion criteria

Studies were excluded if they specifically evaluated post-dural puncture headaches, caffeine as a pain adjunct, effect on ileus or gastrointestinal motility, the risk of arrhythmias from habitual caffeine intake, effects during pregnancy, or were community-based. Studies were also excluded if they investigated the effect of caffeine outside of the intensive care or postoperative hospital setting. Studies that reported on caffeine overdose in the community were not included. Studies were excluded if they reported on individuals under 18 years of age. Animal studies, review articles and editorials that did not contain novel information were excluded.

### Study protocol

The review methodology was conducted according to the Joanna Briggs Institute [20]. Study selection was performed independently by two physician reviewers (MB, VR) at each stage 1 (title and abstract) and stage 2 (full text screening). Any discrepancy was solved by a discussion between the two reviewers and if needed a third reviewer (KL) was available to make the final decision.

Once all articles for inclusion were screened, data was extracted by one of the authors (MB) using the data extraction table. When any information included in a study was unclear, the authors were contacted to provide clarification or further details.

### Analysis

Analysis was primarily descriptive. Given the heterogeneity of the study questions and designs, a quantitative mathematical summary statistic was not calculated. Individual results were summarized where appropriate.

## Results

### Literature search

**Fig. 1:**
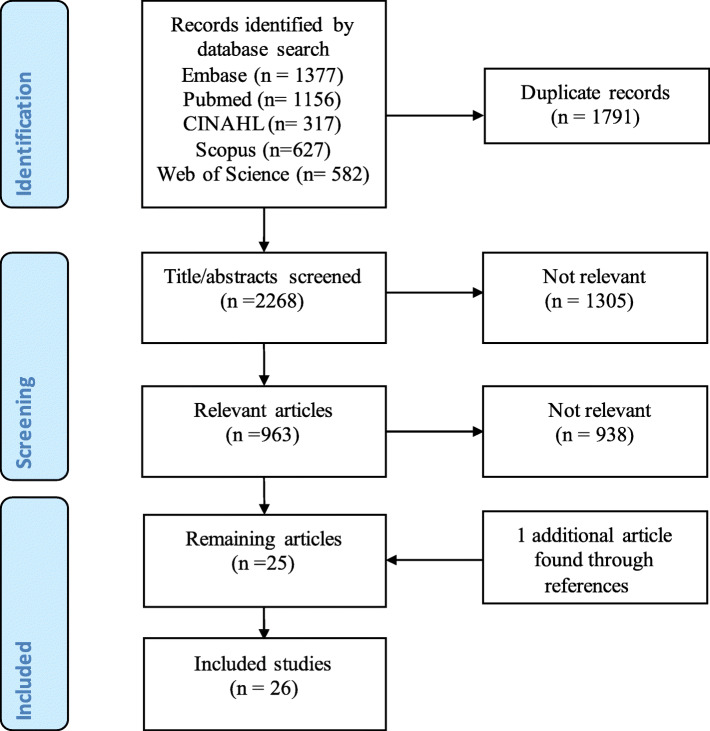
PRISMA flow diagram of article selection

We identified 4059 articles, of which 1791 were duplicates. Of the remaining 2268 unique articles, 1305 were excluded based on their titles and abstracts (Fig. 1). Following full text review of the remaining 963 articles, a further 938 were excluded. The references of the remaining 25 articles were hand-searched for additional publications and one further relevant study was found. The results of the 26 studies are summarized in Table 1.

**Table 1:** Summary of extraction articles

Year	Country	Type of study	Study population	Key findings	Ref
**Caffeine in the intensive care unit**					
*Caffeine withdrawal and administration on the incidence of postoperative headache or delirium*					
2015	USA	Prospective survey	25 intensive care units across 17 institutions	Caffeine intake minimisation was used in 32% of intensive care units as a pharmacological method to reduce delirium	21
*Caffeine withdrawal and administration on induction and emergence from sedation*					
2017	Iran	Prospective RCT	80 patients; 40 coffee, 40 placebo	3.5 g coffee given via nasogastric tube in the mechanically ventilated patients increases the spontaneous respiratory rate and tidal volume but does not significantly affect other respiratory indicators.	22
*Safety and changes associated with caffeine administration*					
1987	Germany	Prospective observational	12 male patients	Quinolones can inhibit the metabolism of caffeine and may cause higher levels of circulating caffeine and side effects	23
1995	Spain	Prospective cohort	Liver impaired 33; normal liver 40	healthy individuals metabolise 3 mg IV caffeine faster than those with liver disease	24
**Caffeine in the perioperative period**					
*Caffeine withdrawal and administration on the incidence of postoperative headache or delirium*					
2017	Greece	Prospective cohort	446 elective surgery patients	In patients with no previous history of headache, caffeine consumption was an additional independent factor for postoperative headache	25
1994	Denmark	Prospective observational	219 elective patients	The risk of postoperative headache was significantly greater in individuals with a daily caffeine intake > 400 mg/day	26
2003	United Kingdom	Prospective observational	208 day-casepatients	Caffeine is not a risk factor for perioperative headache	27
1989	New Zealand	Prospective survey	150 day-case patients	Patient who consume > 200 mg caffeine/day were 3-fold more likely to have a headache postoperatively compared to those who did not	28
1990	Netherlands	Prospective survey	334 GA + 75 LA	There was no difference between incidence of headache between GA or LA alone. Caffeine intake was not a risk factor for developing headache postoperatively.	29
1991	New Zealand	Prospective survey	287 patients undergoing minor elective surgery	postoperative headache is related to caffeine intake and that this relationship is explained at least in part, by a perioperative caffeine withdrawal syndrome	30
1993	USA	Prospective survey	233 surgical outpatients	Among daily caffeine drinkers, those who drank caffeinated beverages on the day of the surgical procedure had a lower incidence of postoperative headaches than did those who abstained (17% versus 28%; *P* < 0.04)	31
1994	Switzerland	Case report	Elective open abdominal surgery for oophorectomy	28F with postoperative headache, hemihypaesthesia, cerebral oedema on CT-Head which resolved with caffeine/ergometrine	32
1995	Switzerland	Prospective RCT	40 patients; 20 caffeine, 20 placebo	Surgical patients who have high caffeine intake were randomised to taking oral caffeine tablets or placebo. No patients on caffeine supplements develop headaches while 10 (50%) on placebo developed headaches which lasted up to 7 days.	33
1997	USA	Prospective RCT	234 elective surgical patients	prophylactic postoperative 200 mg IV caffeine decreased the incidence of headache	34
*Caffeine withdrawal and administration on induction and emergence from anaesthesia*					
2019	United Kingdom	Prospective observational	40 ASA 1 individuals	high daily caffeine intake is associated with lower propofol requirements for induction. We hypothesise that those with high daily caffeine intake have lower arousal levels before surgery, because of a relative caffeine deficit secondary to being nil-by-mouth	39
1984	USA	Prospective RCT	60 patients undergoing CABG	Patients who drank > 3 cups of coffee/day, smoke > 40 cigarettes/day and drank 1–3 oz of alcohol required more fentanyl at induction for their CABG operation	40
1984	Australia	Prospective observational	23 patients + 23 controls	High caffeine intake resulted in worse cognitive functioning post anaesthetic compared to low caffeine intake	46
2011	USA	Case report	Elective tumour resection	The use of 500 mg IV caffeine intraoperatively to ensure the patient is responsive enough to perform intraoperative language mapping. Frequent stimulation-induced seizures thereafter limited further testing.	41
2017	USA	Case report	Elective dental procedure	Use of 60 mg IV caffeine in an 16yo male with trisomy 10 with a history of slow emergence from anaesthesia to speed up emergence from anaesthesia and as a respiratory stimulant	42
2010	Egypt	Prospective RCT	60 patients 30 caffeine, 30 control	Administration of 500 mg IV caffeine decreases the number of patients who developed adverse post extubation respiratory events and hastens recovery from sevoflurane anaesthesia.	45
2018	USA	Prospective RCT	8 male patients	15 mg/kg IV caffeine is able to accelerate emergence from isoflurane anaesthesia in healthy males without any apparent adverse effects	43
2018	USA	Retrospective observational	151 heavily sedated patients in the post-anaesthesia recovery area	Median of 150 mg IV caffeine may enhance the speed of recovery following general anaesthesia without any respiratory or cardiovascular changes	44
*Safety and changes associated with caffeine administration*					
1996	USA	Prospective survey	882 nurses surveyed	85% of responders would withhold caffeine in patients after an acute myocardial infarction as a part of coronary precautions	35
2013	USA	Prospective RCT	30 patients	Ingestion of 102 mg of caffeine (drip coffee) can increase spontaneous voiding post indwelling bladder catheter removal	36
2013	USA	Prospective RCT	62 patients	500 mg IV caffeine given intraoperatively resulted in increased nausea, and there was no difference in postoperative headache, fatigue, time to discharge	37
2018	France	Prospective RCT	110 patients booked for heart valve surgery	400 mg caffeine q8h does not affect postoperative AF but does increase the risk of nausea and vomiting	38

### Characteristics

The 26 articles retrieved were grouped into themes of caffeine in the ICU [21–24] and caffeine in the perioperative setting [25–46]. These were further subdivided into withdrawal headaches or delirium, effect on induction and emergence from sedation and safety/changes associated with caffeine administration. This included 3 case reports [32, 41, 42], 14 studies with 1–199 participants [22–24, 28, 33, 36–40, 43–46], seven studies with 200–500 participants [25–27, 29–31, 34], one with 500–1000 participants [35] and one study that surveyed 25 intensive care units across 17 institutions [21].

### Caffeine use in the intensive care unit

There were four studies investigating the use of caffeine in the intensive care unit, including a national survey and three studies which administered caffeine to ICU patients [21–24]. Dzerba et al performed a national survey of 25 ICUs across 17 institutions in the United States of America to evaluate the delirium screening tools and protocols in place to reduce the incidence of delirium. Afternoon caffeine minimisation was utilised in 32% of ICUs to reduce delirium and improve sleep [21].

One study investigated caffeine administration on induction or emergence from sedation in the ICU. Sadat et al randomised 80 mechanically ventilated ICU patients to either receive 3.5 g of coffee in 100 mL of water or placebo (100 mL of distilled water) at 10 am in the morning [22]. The dose of caffeine administered in this study was unknown. There was a significant increase in spontaneous respiratory rate and tidal volumes at 30 and 60 min in patients who received coffee compared to placebo [22].

Two studies investigated other effects of caffeine. The first study found concomitant administration of quinolones and caffeine in 12 ICU patients could inhibit the metabolism of caffeine resulting in higher plasma levels [23]. The second study was a randomised controlled trial that administered 3 mg/kg caffeine to assess liver function in 33 ICU patients with impaired liver function and 40 with normal liver function [24]. Individuals with impaired liver function had significantly longer elimination of caffeine compared to those with normal liver function. Neither study reported on any adverse events of caffeine administration [23, 24].

### Caffeine use in the perioperative period

There were 22 studies investigating caffeine use in the perioperative period [25–45]. There were 10 studies investigating the effect of caffeine perioperatively and the development of postoperative headache [25–34]. Seven investigated caffeine withdrawal [25–34] and three studies administered oral caffeine to prevent postoperative headache [32–34]. Of the seven prospective studies investigating the effect of caffeine withdrawal, five studies found caffeine withdrawal increased the incidence of postoperative headache [25–34]. Three studies investigated the administration of caffeine to relieve postoperative headache [32–34]. There was one case report of the successful administration of caffeine and ergometrine to relieve a postoperative headache [32]. There were two prospective randomised controlled trials which found prophylactic caffeine administration decreased the incidence of postoperative headache [33, 34].

Eight studies investigated induction and emergence from anaesthesia [39–45]. Individuals who had a high caffeine intake required less propofol for induction [39, 40], but greater opioids for induction for cardiac surgery [46] and lower cognitive scores postoperatively [46]. Furthermore, five studies found the administration of intravenous (IV) caffeine enhanced emergence from anaesthesia [41–45]. Two case reports used IV caffeine to enhance emergence from sedation. The first study in a 52-year old male who received 500 mg IV caffeine to facilitate intraoperative language mapping who was slow to emerge after anaesthesia was ceased [41]. The second case study was a 16-year old male who underwent a dental procedure using sevoflurane anaesthesia who experienced ongoing hypopnea and desaturation up to 90 min after the procedure. Caffeine was used (60 mg or 0.8 mg/kg) and resulted in increased alertness, no further desaturation, increased respiratory rate and tidal volumes [42]. Gouda et al. randomised 60 patients undergoing uvulopalatopharyngoplasty for treatment for obstructive sleep apnoea to either receive 500 mg IV caffeine or saline [45]. Individuals who received caffeine had significantly faster time to extubation and fewer post-extubation respiratory complications (supraglottic obstruction, laryngospasm, reintubation, breath holding, desaturation) [45]. Similar results were demonstrated by Fong et al., who randomised eight patients to receive IV caffeine at 7.5 mg/kg or saline and demonstrated caffeine resulted in significantly faster emergence from isoflurane anaesthesia [43]. This study reported no adverse outcomes from administration of caffeine [43]. Warner et al. performed a retrospective audit of caffeine administration (median dose of 150 mg) in the post-anaesthetic recovery area to increase alertness in 151 heavily sedated patients [44]. There was a significant improvement in sedation scores with no change in respiratory or cardiac outcomes and no reported adverse events [44].

There were four studies that investigated the safety and changes associated with caffeine administration [35–38]. Intraoperative or postoperative caffeine may increase the incidence of postoperative nausea/vomiting [37, 38]. Intraoperative caffeine was found not to affect the incidence of postoperative atrial fibrillation [38] and reduce time to spontaneous voiding post indwelling bladder catheter removal postoperatively [36]. A national survey of 882 nurses found that 85% still practised caffeine restriction in patients after an acute myocardial infarction [35].

## Discussion

In this review we systematically identified only 26 studies that examine caffeine therapeutic use and/or withdrawal in the ICU and perioperative settings. Furthermore, only four studies investigating the use of caffeine in the intensive care unit. This is somewhat surprising considering that withdrawal from caffeine in other settings has been extensively described in the literature for almost 200 years and as an official diagnosis in ICD-10 by the World Health Organisation [19, 47, 48]. Furthermore, given the commonality of use of caffeine products and its broad range of psychoactive and physiologic effects, it may be expected to have significant applications in critical care and perioperative medicine. Despite this, there is a paucity of clinical studies investigating withdrawal or administration of caffeine in the intensive care setting, and use of caffeine to facilitate emergence from anaesthesia or use in the perioperative period.

Onset of caffeine withdrawal occurs as early as 12–24 h post abstinence, with symptoms lasting between 2 to 9 days [19]. It is unsurprising that when chronic caffeine users are admitted to hospital and are required to fast for surgery, they develop a caffeine withdrawal headache postoperatively. Administration of caffeine perioperatively can reduce the incidence of postoperative headache due to caffeine withdrawal. In the intensive care setting, these patients are also likely withdrawing from caffeine. While there is routine screening and management for alcohol or smoking, caffeine consumption or withdrawal is not routinely documented. Currently, there are no studies investigating caffeine withdrawal in the intensive care setting. Although the typical effects of caffeine may not be seen, the effect on circadian rhythm, cognition and mood may be impaired which may contribute to delirium. Caffeine minimisation is one strategy used both in the community and the intensive care setting to reduce the incidence of delirium [21, 49]. In the community, current strategies to reduce delirium include caffeine minimisation after midday. One pilot study across 21 nursing homes and one dementia special care unit found eliminating caffeine intake in the afternoon and evening resulted in significant improvement in sleep scores but no change in agitation/aggression, irritability and aberrant motor behaviour [49]. However, administration of caffeine earlier in the day may help reset and normalise circadian rhythm [50, 51].

Caffeine can facilitate emergence from sedation or anaesthesia. In the intensive care setting, administration of caffeine has been shown to increase spontaneous breathing in intubated patients, which may help wean patients from mechanical ventilation [22]. Postoperatively, caffeine has been used to facilitate emergence from anaesthesia [41–44]. Administration of caffeine to reduce time to emergence from anaesthesia has been demonstrated in animal studies [43, 52, 53]. Increasing emergence from anaesthesia to assist with respiratory drive and return of upper airway tone would be helpful in high risk patients, such as the morbidly obese, individuals with severe obstructive sleep apnoea, or those who are more susceptible to opioid medications with respiratory depression effects. A similar process may occur in adults emerging from anaesthesia that has been suggested in infants, in which caffeine acts centrally on the medullary respiratory centre to increase sensitivity to carbon dioxide demonstrated in infants [10] and may offset the effects from opioids and other sedative medications. The use of caffeine and its derivatives are not routinely used nor are they licensed for the use to facilitate emergence from anaesthesia in adults. Due to the small number of studies included, that used a wide range of caffeine dosing (60 mg to 500 mg), it is unclear what dose will be effective to facilitate emergence from anaesthesia. There is growing interest in the effects of caffeine habits on induction of anaesthesia and in the use of caffeine to increase recovery and emergence from anaesthesia. Individuals with high caffeine intake (> 3 cups/day) may require lower doses of induction agents (including opiates and sedative agents such as propofol), which is thought to be due to caffeine dependence and withdrawal resulting in lower arousal preoperatively [39, 40] . Additionally, individuals with high caffeine intake were shown to have worse cognitive functioning post-anaesthesia, likely from caffeine dependence [46]. None of the studies included administered preoperative caffeine or investigated the risk of awareness with caffeine administration. In non-anaesthetised inviduals who are at rest, the administration of caffeine (3 mg/kg or average of 181 mg) in one small study was found to decrease the proportion of slow delta or theta wave activity as measured by electroencephalogram (EEG) [54]. The effect of 200 mg of caffeine versus placebo on the EEG theta/delta ratio will be further investigated by a randomised, double-blinded clinical trial protocol published in 2016 [55]. No studies to date have investigated the effect of caffeine on the EEG readings in an anaesthetised individual. As caffeine is a central nervous system stimulant, there is a risk that early administration of caffeine could increase awareness during anaesthesia. The use of caffeine in the ICU setting has the benefit of reducing excessive daytime somnolence and removing barriers to participate in daytime physiotherapy.

The secondary beneficial effects of caffeine administration could be exploited in the ICU setting. Gastrointestinal dysmotility is a common problem in the intensive care patients, compounded by opiates, surgery, sepsis and electrolyte abnormalities [56]. Caffeine has been shown to effectively improve gastrointestinal motility with no significant side effect [57]. Historically, caffeine intake has been limited in the perioperative period due to the risk of vasospasm and risk of cardiac events [58, 59] . However, caffeine use after an acute myocardial infarct may be reduce the risk of cardiovascular mortality [60, 61] and does not increase the risk of atrial fibrillation after cardiac surgery [38]. At rest, caffeine appears to promote generalised vasodilation and does not affect digital microvascular perfusion [62, 63], however it does result in reduced cerebral blood flow [64, 65]. Additionally, there are psychological benefits due to the socio-cultural aspects associated with coffee and caffeine [66]. Future studies investigating the use of caffeine in the ICU setting should be aware of these secondary effects particular in patients who have undergone neurological or plastic reconstructive procedures or have had a recent stroke. Additionally, future studies need to consider the significant interindividual variability with respect to caffeine metabolism by CYP 450 1A1/2 that resulting in a halflife from 3 to 10 h [7, 67]. This may also explain the large variations in daily caffeine intake and differences in pharmacodynamics present in the population. These differences may be more pronounced in individuals who have a high caffeine intake.

As a scoping review, the major limitation is the inability to perform systematic analysis between groups due to the heterogeneity of papers and themes found. Advantages of this review include no language limitation, inclusion of grey research (i.e. conference abstracts) and a thorough search using a comprehensive search strategy designed with the assistance of an expert medical librarian.

## Conclusion

In conclusion, we identified some areas of caffeine use but this is largely an under investigated area. Caffeine withdrawal due to hospitalisation occurs rapidly and patients can develop withdrawal symptoms including headache. Administration of caffeine or coffee supplementation has been shown to be safe, can result in faster emergence from anaesthesia. This scoping review has highlighted gaps in the literature regarding the use of caffeine in the intensive care unit and in the perioperative period. Studies examining mitigation of withdrawal effects of caffeine use, optimal dosing, preferred route of administration (i.e. parenteral versus oral) and therapeutic use of caffeine as adjunctive therapies for pain management and delirium are a priority.

## Supplementary Information

**Additional file 1: Appendix.** Search criteria.

## Data Availability

Not applicable.

## Abbreviations

ICU: Intensive Care Unit
IV: Intravenous
