# Supplementary material for: Use of therapeutic caffeine in acute care postoperative and critical care settings: a scoping review
Source: BMC Anesthesiol. 2021 Mar 31;21:100. doi: 10.1186/s12871-021-01320-x (PMC8011218; doi:10.1186/s12871-021-01320-x)
Supplement: Supplementary file 1 — Additional file 1: Appendix. Search criteria. [file 12871_2021_1320_MOESM1_ESM.docx]

Appendix 1: Search criteria

**PubMed (new search interface, changing from “Best Match” algorithm default option to “Publication date”**, with Medical Subject Headings)**:** 1156 results

(“caffeine”[tiab] OR “coffee”[tiab] OR "Caffeine"[Mesh] OR "Coffee"[Mesh]) AND (“anesthesia recovery period”[tiab] OR “anaesthesia recovery period”[tiab] OR “postoperative”[tiab] OR “post-operative”[tiab] OR “post operative”[tiab] OR “postoperatively”[tiab] OR “post-operatively”[tiab] OR “post operatively”[tiab] OR "Postoperative Period"[Mesh] OR "Postoperative Complications"[Mesh] OR “post-anaesthetic”[tiab] OR “post-anesthetic”[tiab] OR “postanaesthetic”[tiab] OR “postanesthetic”[tiab] OR “intensive care”[tiab] OR "Intensive Care Units"[Mesh] OR “critical care”[tiab] OR "Critical Care"[Mesh] OR "Anesthesia, General"[Mesh] OR “anesthesia”[tiab] OR “anaesthesia”[tiab])

**Embase** (including Emtree)**:** 1377 results

(“caffeine”:ti,ab OR “coffee”:ti,ab OR 'caffeine'/de OR 'coffee'/de) AND (“anesthesia recovery period”:ti,ab OR “anaesthesia recovery period”:ti,ab OR “postoperative”:ti,ab OR “post-operative”:ti,ab OR “post operative”:ti,ab OR “postoperatively”:ti,ab OR “post-operatively”:ti,ab OR “post operatively”:ti,ab OR 'postoperative period'/de OR 'postoperative complication'/de OR “post-anaesthetic”:ti,ab OR “post-anesthetic”:ti,ab OR “postanaesthetic”:ti,ab OR “postanesthetic”:ti,ab OR “intensive care”:ti,ab OR 'intensive care unit'/de OR “critical care”:ti,ab OR 'intensive care'/de OR 'general anesthesia'/de OR “anesthesia”:ti,ab OR “anaesthesia”:ti,ab)

**CINAHL Complete** (including CINAHL Subject Headings)**:** 317 results

(TI (“caffeine” OR “coffee”) OR AB (“caffeine” OR “coffee”) OR (MH "Caffeine") OR (MH "Coffee")) AND (TI (“anesthesia recovery period” OR “anaesthesia recovery period” OR “postoperative” OR “post-operative” OR “post operative” OR “postoperatively” OR “post-operatively” OR “post operatively” OR “post-anaesthetic” OR “post-anesthetic” OR “postanaesthetic” OR “postanesthetic” OR “intensive care” OR “critical care” OR “anesthesia” OR “anaesthesia”) OR AB (“anesthesia recovery period” OR “anaesthesia recovery period” OR “postoperative” OR “post-operative” OR “post operative” OR “postoperatively” OR “post-operatively” OR “post operatively” OR “post-anaesthetic” OR “post-anesthetic” OR “postanaesthetic” OR “postanesthetic” OR “intensive care” OR “critical care” OR “anesthesia” OR “anaesthesia”) OR (MH "Postoperative Period") OR (MH "Postoperative Complications+") OR (MH "Intensive Care Units+") OR (MH "Critical Care+") OR (MH "Anesthesia, General+"))

**Scopus** (Title and Abstract search) 627 results

TITLE-ABS((“caffeine” OR “coffee”) AND (“anesthesia recovery period” OR “anaesthesia recovery period” OR “postoperative” OR “post-operative” OR “post operative” OR “postoperatively” OR “post-operatively” OR “post operatively” OR “post-anaesthetic” OR “post-anesthetic” OR “postanaesthetic” OR “postanesthetic” OR “intensive care” OR “critical care” OR “anesthesia” OR “anaesthesia”))

**Web of Science** (Topic search) 582 results

TS=((“caffeine” OR “coffee”) AND (“anesthesia recovery period” OR “anaesthesia recovery period” OR “postoperative” OR “post-operative” OR “post operative” OR “postoperatively” OR “post-operatively” OR “post operatively” OR “post-anaesthetic” OR “post-anesthetic” OR “postanaesthetic” OR “postanesthetic” OR “intensive care” OR “critical care” OR “anesthesia” OR “anaesthesia”))
